# Clinical implications of cardiac troponin-I in patients with hypertensive crisis visiting the emergency department

**DOI:** 10.1080/07853890.2022.2034934

**Published:** 2022-02-03

**Authors:** Woohyeun Kim, Byung Sik Kim, Hyun-Jin Kim, Jun Hyeok Lee, Jinho Shin, Jeong-Hun Shin

**Affiliations:** a Department of Internal Medicine, Division of Cardiology, Hanyang University Seoul Hospital, Hanyang University College of Medicine, Seoul, Republic of Korea; b Division of Cardiology, Department of Internal Medicine, Hanyang University Guri Hospital, Hanyang University College of Medicine, Guri, Republic of Korea; c Department of Biostatistics, Yonsei University Wonju College of Medicine, Wonju, Republic of Korea

**Keywords:** Cardiac troponin, hypertensive crisis, mortality, emergency department

## Abstract

**Objectives:**

Cardiac troponin-I (cTnI) is a representative marker of myocardial injury. Elevation of cTnI is frequently observed in patients with hypertensive crisis, but few studies have examined its prognostic significance in hypertensive crisis. We aimed to determine whether cTnI could predict all-cause mortality in patients with hypertensive crisis visiting the emergency department (ED).

**Methods:**

This observational study included patients aged ≥18 years who visited an ED between 2016 and 2019 for hypertensive crisis, defined as systolic blood pressure (BP) ≥180 mmHg and/or diastolic BP ≥110 mmHg. Among 6467 patients, 3938 who underwent a cTnI assay were analysed.

**Results:**

Among the 3938 patients, 596 (15.1%) had cTnI levels above the 99th percentile upper reference limit (elevated cTnI >40 ng/L) and 600 (15.2%) had cTnI levels between the detection limit (≥10 ng/L) and the 99th percentile upper reference limit (detectable cTnI). The 3-year all-cause mortality in the elevated, detectable and undetectable cTnI groups were 41.6%, 36.5% and 12.8%, respectively. After adjusting for confounding variables, elevated cTnI patients (adjusted hazard ratio [HR], 2.01; 95% confidence interval [CI], 1.61–2.52) and detectable cTnI patients (adjusted HR, 1.64; 95% CI, 1.32–2.04) showed a significantly higher risk of 3-year all-cause mortality than did patients with undetectable cTnI.

**Conclusions:**

In patients with hypertensive crisis, elevated cTnI levels provide useful prognostic information and permit the early identification of patients with an increased risk of death. Moreover, putatively normal but detectable cTnI levels also significantly correlated with a higher risk of all-cause mortality. Intensive treatment and follow-up strategies are needed for patients with hypertensive crisis with elevated and detectable cTnI levels.

**Key messages**
1. Cardiac troponin-I level was an independent prognostic factor for all-cause mortality in patients with hypertensive crisis.
2. Detectable but normal range cardiac troponin-I, which was considered clinically insignificant, also had a prognostic impact on all-cause mortality comparable to elevated cardiac troponin-I levels.

## Introduction

Hypertensive crisis is a clinical condition commonly encountered among patients visiting the emergency department (ED). Patients with hypertensive crisis reportedly account for 3.2% of patients visiting the ED and approximately 1–2% of hypertensive patients experience hypertensive crisis throughout their life [1].

In clinical practice, the treatment for hypertensive crisis focuses on preventing or minimising acute hypertension-mediated organ damage (HMOD) by appropriately controlling blood pressure (BP) [2–4]. This presupposes that appropriate screening and pre-emptive management of individuals at risk for acute HMOD could improve the clinical outcomes of these patient groups. However, there is little data on markers related to acute HMOD and its prognosis in hypertensive crisis.

Cardiac troponin-I (cTnI) is a cardiac-specific protein with a very high sensitivity and specificity for myocardial injury [5,6]. It has been reported that it can predict major adverse cardiovascular events not only in patients with cardiovascular disease, but also in the general population [5,7]. Elevation of cardiac troponin is frequently observed in patients with hypertensive crisis, but few studies have investigated the prognostic significance of this elevation in hypertensive crisis. This study aimed to evaluate the clinical implications of cTnI levels on all-cause mortality in patients with hypertensive crisis who visited the ED.

## Materials and methods

### Study population

**Figure 1.**
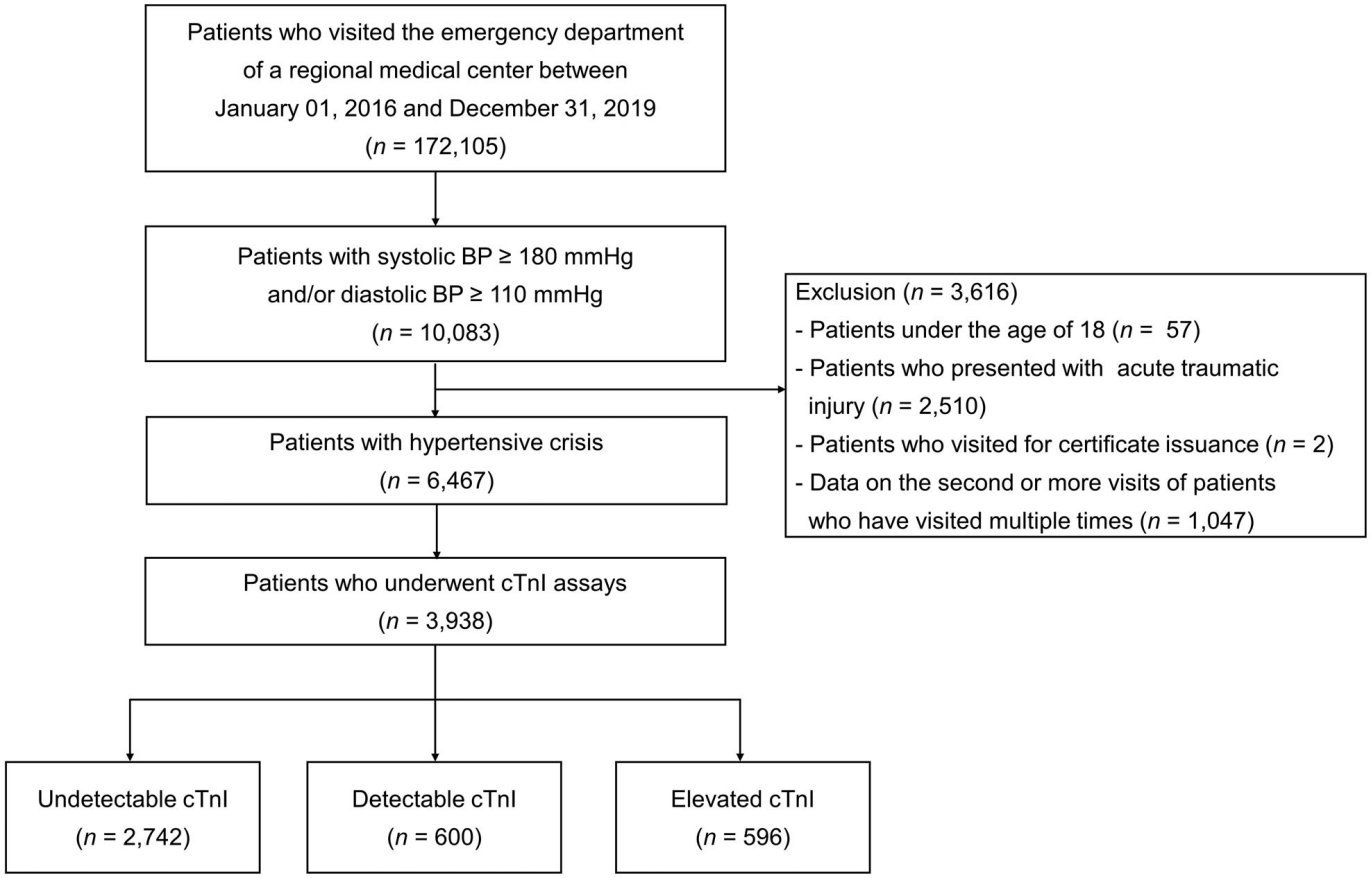
Flow diagram illustrating patients with hypertensive crisis who were included in this study. cTnI, cardiac troponin-I.

In this observational study, we analysed a total of 172,105 patients who visited the ED of Hanyang University Guri Hospital from January 2016 to December 2019. Among 10,083 patients with an initial triage systolic BP ≥ 180 mmHg and/or diastolic BP ≥ 110 mmHg, patients under 18 years of age, those with acute trauma and those who visited for certificate issuance were excluded. Only data from the first visit were included in patients with multiple visits to the ED. Among the 6467 patients with hypertensive crisis, 3938 who underwent cTnI assays were analysed (Figure 1). Patients with hypertensive crisis were further divided into hypertensive emergency and hypertensive urgency according to the presence or absence of acute HMOD. Acute HMOD was defined by the presence of one of the following conditions: hypertensive encephalopathy, cerebral infarction, intracerebral haemorrhage, retinopathy, acute heart failure, acute coronary syndrome, acute renal failure and aortic dissection [8].

### Data collection and outcomes

Data were collected using the electronic medical records. The detailed study design and definitions of comorbidities in our study have been published previously [8,9]. Briefly, baseline data for enrolled patients were obtained at the index visit of ED, and events during the follow-up periods were obtained until death from any cause or the end of the study (March 2021). The incidence and timing of death were extracted from the National Health Insurance Service in South Korea. The study was approved by the Institutional Review Board of Hanyang university Guri hospital and was conducted in accordance with the Declaration of Helsinki.

### Serum cardiac troponin-I assay

Serum levels of cTnI were measured using an immunoassay system (AccuTnI + 3; Beckman Coulter Inc., Brea, CA). The upper reference limit (99th percentile) for cTnI among healthy adults is 40 ng/L and the limit of detection is <10 ng/L [10]. In our study, we divided the study population into three groups according to cTnI level. Patients with values exceeding the upper reference limit (>40 ng/L) were identified as the “elevated cTnI” group, while those with values less than the limit of detection (<10 ng/L) were identified as the “undetectable cTnI” group. Patients not included in either of those groups were classified into the “detectable cTnI” group.

### Statistical analysis

Data are presented as means (standard deviation) for continuous variables and as frequency (percentage) for categorical variables. The baseline characteristics were compared using the one-way analysis of variance/Kruskal–Wallis test followed by Tukey’s post hoc test/Dunn’s multiple comparison or the chi-squared/Fisher’s exact test, as appropriate. The 3-year all-cause mortality estimates were computed using the Kaplan–Meier survival analysis, with group comparisons made using the log-rank test. The independent predictive value of cTnI on 3-year all-cause mortality was determined using a Cox proportional hazards regression model with consideration of other clinically relevant variables, including baseline characteristics (age, sex, systolic blood pressure [SBP] and diastolic blood pressure [DBP]), comorbidities (hypertension, diabetes mellitus, ischaemic stroke, haemorrhagic stroke, coronary artery disease (CAD) and chronic kidney disease) and components of HMOD (estimated glomerular filtration rate, cardiomegaly on chest radiography, left ventricular hypertrophy on electrocardiography and myocardial ischaemia on electrocardiography). Additionally, we performed a subgroup analysis with multivariable Cox regression analysis using the same adjusting variables, stratified by covariates including age (≥65 or <65 years), hypertensive emergency or hypertensive urgency, presentation with or without acute myocardial infarction (AMI), and presence or absence of a history of CAD. Hazard ratios (HRs) and respective 95% confidence intervals (CIs) were calculated. All tests were two-tailed, and the statistical significance was set at *p* < .05. All analyses were performed using the Statistical Analysis Software package (SAS version 9.4; SAS Institute, Cary, NC).

## Results

### Baseline characteristics

A total of 3938 patients were enrolled in the final analysis, and follow-up data for up to 5.2 years were analysed. The median follow-up period was 3.0 years (interquartile range, 2.1–4.0 years). The baseline characteristics of the patients according to cTnI levels are shown in Table 1. Among these patients, 596 (15.1%) had elevated cTnI (>40 ng/L), 600 (15.2%) had detectable cTnI (≥10 and ≤40 ng/L), and 2742 (69.6%) had undetectable cTnI (<10 ng/L). Mean age was greater in patients with detectable cTnI (68.8 ± 15.0 *vs.* 71.2 ± 14.5 *vs.* 62.2 ± 15.7, *p* < .001). Patients with elevated cTnI levels had the lowest proportion of female individuals (42.3% *vs.* 49.8% *vs.* 50.7%, *p* < .001). Acute HMOD was predominantly observed in patients with elevated cTnI levels (70.0% *vs.* 45.8% *vs.* 30.5%, *p* < .001). Patients with elevated cTnI and detectable cTnI had more cardiovascular risk factors, including hypertension, diabetes mellitus, ischaemic stroke, CAD, heart failure, chronic kidney disease and end-stage renal disease than patients with undetectable cTnI levels. Presenting SBP was higher in patients with elevated cTnI and detectable cTnI levels than in patients with undetectable cTnI levels (196 ± 24.9 *vs.* 197 ± 23.8 *vs.* 191 ± 20.9, *p* < .001), but there was no significant difference in DBP between the groups. In the ED, patients with elevated cTnI and detectable cTnI levels also had worse laboratory findings, including serum creatinine, estimated glomerular filtration rate, B-type natriuretic peptide, D-dimer and haemoglobin levels than patients with undetectable cTnI levels. In addition, cardiomegaly and congestion on chest radiography, left ventricular hypertrophy, myocardial ischaemia and atrial fibrillation on electrocardiography were more frequently observed in patients with elevated cTnI and detectable cTnI levels than in patients with undetectable cTnI levels.

**Table 1.** Baseline characteristics according to cardiac troponin-I levels.

	All patients (*n* = 3938)	Elevated cTnI^a^ (*n* = 596)	Detectable cTnI^b^ (*n* = 600)	Undetectable cTnI^c^ (*n* = 2742)	*p* Value
Age, mean (SD)	64.6 (15.8)	68.8 (15.0)^†^*	71.2 (14.5) ↑	62.2 (15.7)	<.001
Female sex, *n* (%)	1941 (49.3)	252 (42.3)	299 (49.8)	1390 (50.7)	<.001
Body mass index, kg/m^2^	24.31 (4.31)	24.04 (4.66)^†^	23.58 (4.36) ↑	24.60 (4.15)	<.0001
Medical history, *n* (%)					
Hypertension	2258 (58.5)	405 (69.0)	409 (68.9)	1444 (53.8)	<.001
Diabetes mellitus	1160 (30.2)	245 (41.9)	215 (36.4)	700 (26.2)	<.001
Dyslipidaemia	402 (10.5)	62 (10.7)	56 (9.51)	284 (10.7)	.683
Ischaemic stroke	361 (9.5)	79 (13.6)	75 (12.7)	207 (7.8)	<.001
Haemorrhagic stroke	112 (2.9)	16 (2.8)	19 (3.2)	77 (2.9)	.893
Coronary artery disease	428 (11.2)	89 (15.3)	75 (12.6)	264 (10.0)	<.001
Peripheral artery disease	41 (1.1)	8 (1.4)	10 (1.7)	23 (0.9)	.16
Heart failure	203 (5.3)	84 (14.5)	60 (10.2)	59 (2.2)	<.001
Chronic kidney disease	380 (9.9)	158 (27.1)	120 (20.2)	102 (3.9)	<.001
End-stage renal disease	182 (4.8)	95 (16.3)	61 (10.4)	26 (1.0)	<.001
Social history, *n* (%)					
Cigarette smoking	816 (29.4)	164 (32.4)	99 (21.3)	553 (30.6)	<.001
Alcohol consumption	967 (34.4)	133 (26.2)	115 (24.6)	719 (39.1)	<.001
Triage vitals, mean (SD)					
SBP, mmHg	193 (22.1)	196 (24.9)^†^	197 (23.8) ↑	191 (20.9)	<.001
DBP, mmHg	107 (18.0)	107 (20.1)	105 (20.8)	107 (16.8)	.229
Laboratory tests					
Mean serum creatinine, mg/dL (SD)	1.34 (1.79)	2.52 (2.96)^†^*	1.91 (2.35) ↑	0.96 (0.99)	<.001
Mean eGFR, mL/min/1.73 m^2^ (SD)	76.5 (31.1)	54.0 (34.8)^†^*	60.3 (32.8) ↑	85.6 (24.9)	<.001
BNP, pg/mL (SD)	379 (791)	1000 (1300)^†^*	478 (641) ↑	110 (250)	<.001
D-dimer, mg/L (SD)	791 (2980)	1530 (4260)^†^	1120 (3790) ↑	479 (2070)	<.001
Hb, g/dL (SD)	13.4 (2.2)	12.5 (2.7)^†^	12.6 (2.5) ↑	13.7 (1.9)	<.001
Urinary analysis done, *n* (%)	2654 (67.4)	446 (74.8)	431 (71.8)	1777 (64.8)	<.001
Proteinuria^d^, *n* (%)	918 (34.6)	283 (63.5)	225 (52.4)	410 (23.1)	<.001
Chest X-ray done, *n* (%)	3773 (95.8)	571 (95.8)	579 (96.5)	2623 (95.7)	.649
Cardiomegaly, *n* (%)	538 (14.2)	107 (18.8)	112 (19.3)	319 (12.1)	<.001
Congestion/fluid overload, *n* (%)	273 (7.2)	140 (24.6)	77 (13.3)	56 (2.1)	<.001
ECG done, *n* (%)	3738 (94.9)	572 (96.0)	578 (96.3)	2588 (94.4)	.0641
LVH, *n* (%)	461 (12.4)	90 (15.8)	102 (17.7)	269 (10.4)	<.001
Myocardial ischaemia, *n* (%)	288 (7.7)	122 (21.4)	45 (7.8)	121 (4.7)	<.001
Atrial fibrillation, *n* (%)	222 (6.0)	64 (11.2)	54 (9.4)	104 (4.0)	<.001
Acute HMOD, *n* (%)	1527 (38.8)	417 (70.0)	275 (45.8)	835 (30.5)	<.001
Patients taking no antihypertensive drug, *n* (%)	626 (27.7)	115 (28.4)	106 (25.9)	405 (28.0)	.396

Data are presented as n (%) or mean (SD), as appropriate. SD: standard deviation; cTnI: cardiac troponin-I; SBP: systolic blood pressure; DBP: diastolic blood pressure; eGFR: estimated glomerular filtration rate; BNP: B-type natriuretic peptide; Hb: haemoglobin; ECG: electrocardiography; LVH: left ventricular hypertrophy; HMOD: hypertension-mediated organ damage

^a^Elevated cTnI is defined as a cardiac troponin-I level >40 ng/L.

^b^Detectable cTnI is defined as a cardiac troponin-I level ≥10 and ≤40 ng/L.

^c^Undetectable cTnI is defined as a cardiac troponin-I level <10 ng/L.

^d^Proteinuria was defined as a dipstick urinalysis result ≥ 1+.

†Post hoc *p*: Elevated cTnI group versus undetectable cTnI group, statistically significant (*p* < .05).

*Post hoc *p*: Elevated cTnI group versus detectable cTnI group, statistically significant (*p* < .05).

↑Post hoc *p*: Detectable cTnI group versus undetectable cTnI group, statistically significant (*p* < .05).

### Outcomes of the index visit and during the follow-up period

**Figure 2.**
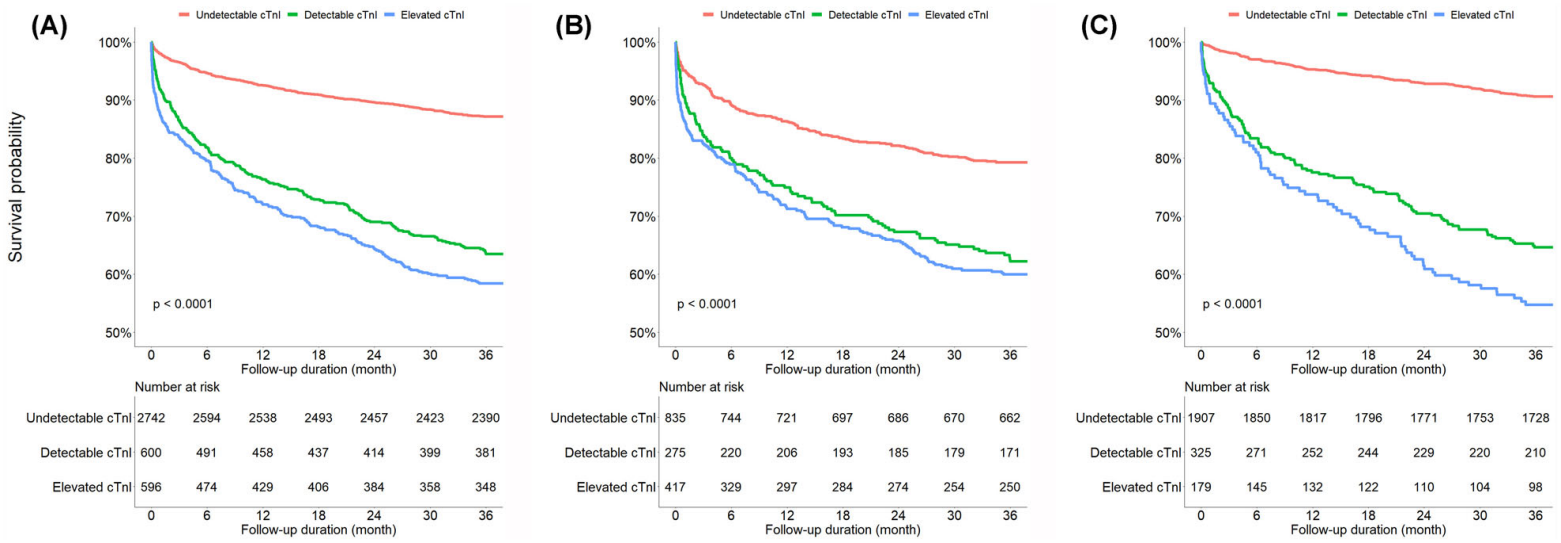
Kaplan–Meier curves comparing 3-year all-cause mortality between groups according to cardiac troponin-I. (A) All patients. (B) Patients with hypertensive emergency. (C) Patients with hypertensive urgency. cTnI: cardiac troponin-I.

Patients with elevated cTnI and detectable cTnI levels were more likely to be admitted than patients with undetectable cTnI levels (84.4% *vs.* 70.0% *vs.* 48.1%, *p* < .001). Rates of ED revisit and readmission were higher in patients with elevated cTnI and detectable cTnI levels than in those with undetectable cTnI levels. One-month, three-month, one-year and three-year mortality rates were highest in patients with elevated cTnI levels, and surprisingly, were also significantly higher in patients with detectable cTnI levels than in those with undetectable cTnI levels (Table 2). The 3-year all-cause mortality in the elevated cTnI group, detectable cTnI group, and undetectable cTnI group was 41.6%, 36.5% and 12.8%, respectively. Time-to-event analysis using the Kaplan–Meier method showed the highest rate of death in patients with elevated cTnI levels and the lowest rate of death in patients with undetectable cTnI levels (Figure 2(A)). Similar trends were observed in the subgroups according to the presence of acute HMOD (Figure 2(B,C)). After adjusting for age, sex, SBP, DBP, comorbidities and components of HMOD, patients with elevated cTnI (adjusted HR 2.01; 95% CI, 1.61–2.52) and patients with detectable cTnI (adjusted HR 1.64; 95% CI, 1.32–2.04) showed a significantly higher risk of 3-year all-cause mortality than patients with undetectable cTnI (Table 3).

**Table 2.** Outcomes of the index visit to the emergency department and during the follow-up period according to cardiac troponin-I levels.

	All patients (*n* = 3938)	Elevated cTnI^a^ (*n* = 596)	Detectable cTnI^b^ (*n* = 600)	Undetectable cTnI^c^ (*n* = 2742)	*p* Value
Outcomes of the index visit to the ED, *n* (%)					
Admission	2242 (56.9)	503 (84.4)	420 (70.0)	1319 (48.1)	<.001
Discharge	1261 (32.0)	40 (6.71)	119 (19.8)	1102 (40.2)	<.001
Discharge against medical advice	432 (11.0)	50 (8.39)	61 (10.2)	321 (11.7)	.050
Death in the emergency department	4 (0.1)	4 (0.7)	0 (0)	0 (0)	<.001
Revisit to ED, *n* (%)					
1-month revisit	275 (9.0)	41 (8.3)	60 (12.3)	174 (8.4)	.021
3-months revisit	520 (17.1)	89 (18.1)	116 (23.9)	315 (15.3)	<.001
1-year revisit	937 (30.8)	155 (31.4)	189 (38.9)	593 (28.7)	<.001
Readmission, n (%)					
1-month readmission	189 (6.2)	28 (5.7)	34 (7.0)	127 (6.1)	.678
3-months readmission	291 (9.5)	54 (10.9)	56 (11.5)	181 (8.8)	.093
1-year readmission	463 (15.2)	81 (16.4)	99 (20.3)	283 (13.7)	<.001
Mortality, *n* (%)					
1-month mortality	180 (4.6)	76 (12.8)	49 (8.2)	55 (2.1)	<.001
3-months mortality	274 (7.0)	98 (16.4)	81 (13.5)	95 (3.5)	<.001
1-year mortality	513 (13.0)	167 (28.0)	142 (23.7)	204 (7.4)	<.001
3-year mortality	819 (20.8)	248 (41.6)	219 (36.5)	352 (12.8)	<.001

Data are presented as n (%). cTnI: cardiac troponin-I; ED: emergency department

^a^Elevated cTnI is defined as a cardiac troponin-I level >40 ng/L.

^b^Detectable cTnI is defined as a cardiac troponin-I level ≥10 and ≤40 ng/L.

^c^Undetectable cTnI is defined as a cardiac troponin-I level <10 ng/L.

**Table 3.** The 3-year all-cause mortality rates and hazard ratios for mortality according to cardiac troponin-I levels among patients with hypertensive crisis.

	3-year mortality	Unadjusted HR (95% CI)	Model 1^a^ (95% CI)	Model 2^b^ (95% CI)	Model 3^c^ (95% CI)
Undetectable cTnI^d^	12.8%	REF	REF	REF	REF
Detectable cTnI^e^	36.5%	3.31 (2.79-3.91)	2.14 (1.80-2.54)	1.98 (1.66-2.36)	1.64 (1.32-2.04)
Elevated cTnI^f^	41.6%	4.00 (3.40-4.70)	3.02 (2.57-3.57)	2.62 (2.20-3.13)	2.01 (1.61-2.51)

HR, hazard ratio; CI, confidence interval; cTnI, cardiac troponin-I.

^a^Model 1: Adjustment for age, and sex.

^b^Model 2: Adjustment for age, sex, systolic blood pressure, diastolic blood pressure, and comorbidities (hypertension, diabetes mellitus, ischaemic stroke, haemorrhagic stroke, coronary artery disease, and chronic kidney disease).

^c^Model 3: Adjustment for age, sex, systolic blood pressure, diastolic blood pressure, comorbidities (hypertension, diabetes mellitus, ischaemic stroke, haemorrhagic stroke, coronary artery disease, and chronic kidney disease), and components of hypertension-mediated organ damage (estimated glomerular filtration rate, cardiomegaly on chest radiography, left ventricular hypertrophy on electrocardiography, and myocardial ischaemia on electrocardiography).

^d^Undetectable cTnI is defined as a cardiac troponin-I level <10 ng/L.

^e^Detectable cTnI is defined as a cardiac troponin-I level ≥10 and ≤40 ng/L.

^f^Elevated cTnI is defined as a cardiac troponin-I level >40 ng/L.

**Figure 3.**
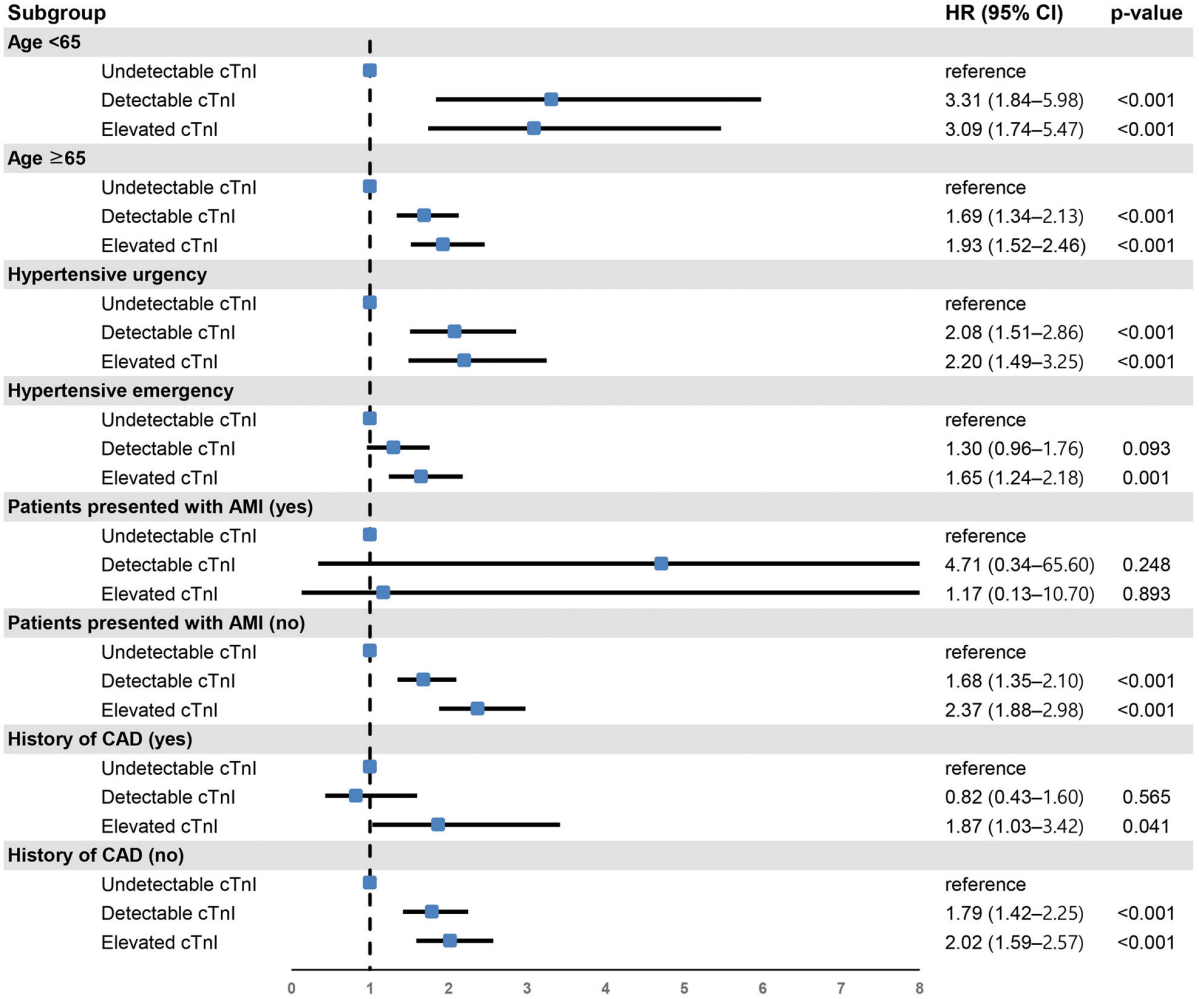
Risk of 3-year all-cause mortality according to cardiac troponin-I in subgroups. cTnI, cardiac troponin-I. Hazard ratios were adjusted for age, sex, systolic blood pressure, diastolic blood pressure, comorbidities (hypertension, diabetes mellitus, ischaemic stroke, haemorrhagic stroke, coronary artery disease and chronic kidney disease) and components of hypertension-mediated organ damage (estimated glomerular filtration rate, cardiomegaly in chest radiography, left ventricular hypertrophy in electrocardiography and myocardial ischaemia in electrocardiography).

Additionally, we performed a subgroup analysis stratified by age (≥65 or <65 years), hypertensive emergency or hypertensive urgency, presentation with or without AMI, and presence or absence of a history of CAD. It showed that the HR and 95% CI for all-cause mortality of the three categories (undetectable cTnI, detectable cTnI and elevated cTnI) were similar in all subgroups except for patients with hypertensive emergency, those presenting with AMI and the presence of a history of CAD. The impact of cTnI on the risk of 3-year all-cause mortality was also prominent in patients without AMI and without a history of CAD (Figure 3).

## Discussion

We investigated the clinical implications of cTnI levels on all-cause mortality in patients with hypertensive crisis who visited the ED. The main results of this study were as follows: in the setting of hypertensive crisis, (1) patients with elevated cTnI had a higher risk of mortality independent of other clinically relevant variables, including baseline characteristics, comorbidities and components of HMOD; (2) this prognostic significance of elevated cTnI was consistently observed regardless of the presence of acute HMOD, age (<65 or ≥65 years), and a history of CAD, except for patients presenting with AMI; and (3) detectable cTnI, which was considered clinically insignificant, had a prognostic impact on all-cause mortality comparable to elevated cTnI levels.

Cardiac troponin is a component of the contractile apparatus of cardiomyocytes expressed almost exclusively in the heart and is the preferred biomarker for the evaluation of myocardial injury [6]. Cardiac troponin values indicate myocardial necrosis in acute coronary syndrome, but they are not disease-specific and may be elevated in various ischaemic, non-ischaemic and extra-cardiac conditions [11]. Furthermore, cardiac troponin concentrations may directly reflect various pathophysiological processes, such as myocyte necrosis and apoptosis. In a hypertensive crisis setting, left ventricular wall stress due to an increase in afterload and subendocardial ischaemia due to an accompanying catecholamine surge can elevate cardiac troponin levels, even in the absence of CAD [5,12].

It has been suggested that the assessment of cardiac troponin may be suitable for predicting the first and subsequent adverse events not only in patients with CAD but also in the general population [6,7,13–16]. A large number of studies have shown that elevation of the cardiac troponin concentration, including concentrations not above the 99th percentile upper reference limit, is a strong risk marker for death, the development of heart failure, ischaemic heart disease and other non-ischaemic cardiac diseases [17–24]. However, few studies have examined the prognostic implications of cardiac troponin levels in patients with hypertensive crisis. Pattanshetty et al. analysed 236 patients with hypertensive crisis, in which the risk of major adverse cardiovascular events was higher in patients with elevated cTnI levels (more than the upper reference limit) than in patients with normal cTnI levels [25]. In contrast, according to the results of a retrospective analysis of 567 patients with hypertensive emergency by Afonso et al., cTnI elevation was not associated with mortality [26]. Because these studies identified patients by diagnostic code or primary diagnosis, it is likely that some patients with hypertensive crisis were not included. Our study showed that cTnI is associated with an increased risk of all-cause mortality at 3 years and that it is a strong predictor of mortality regardless of the presence of acute HMOD. In addition, unlike previous studies, we analysed patients with non-elevated cTnI levels (less than the upper reference limit) by dividing them into detectable cTnI (within normal range, but detectable value) and undetectable cTnI (undetectable value) groups. Surprisingly, patients with detectable cTnI levels showed a higher risk of all-cause mortality than patients with undetectable cTnI levels, comparable to those with elevated cTnI levels.

The most important use of cTnI testing is to identify patients suspected of having AMI, and AMI may be a type of acute HMOD that results from a hypertensive crisis. Therefore, it is of great interest to understand whether the prognostic value of cTnI observed in our study has advantages in addition to AMI diagnosis. Interestingly, the prognostic implications were consistent when subgroup analyses were performed, excluding patients presenting with AMI at the time of ED visit and excluding patients with a history of CAD. These results provide more robust evidence for the prognostic value of cTnI in patients with hypertensive crisis.

Cumulatively, our findings showed that additional risk stratification is needed to identify patients at higher risk for cardiovascular events or mortality and provide aggressive and focalized preventive treatment for patients with hypertensive crisis visiting an ED. In this respect, a routine assessment of cardiac troponin levels to rule out asymptomatic myocardial injuries and prognostic risk stratification would have clinical implications. Future research regarding biomarkers for predicting cardiovascular events or mortality in patients with hypertensive crisis is needed.

This study has several strengths. First, to the best of our knowledge, this is the first large-scale study to report the prognostic significance of cardiac troponin in patients with hypertensive crisis visiting the ED. Second, although our study was conducted with a single-centre retrospective design, since all patients were surveyed based on BP at the time of ED visit using the National Emergency Department Information System (NEDIS) data, there is little chance that patients with hypertensive crisis are dropped from the study population. In Korea, the information of all patients who visit emergency medical institutions is automatically transferred from each hospital to NEDIS, a central government server. The system collects data, including initial vital signs and demographic and baseline clinical characteristics. In this respect, this study reflects the actual state of hypertensive crisis in EDs in South Korea.

This study had several limitations. First, as this study was a retrospective observational study, caution is required in interpretation of causal relationships. Although the study was based on available medical records, retrospective data, such as the clinical history, laboratory and diagnostic tests performed in ED were insufficient. Similarly, the medical history of CAD and diagnosis of AMI were determined based on the information recorded on the chart through an interview at the time of the ED visit, and the diagnostic accuracy and specificity of this information cannot be guaranteed. Second, because the data about antihypertensive medication after ED visits are limited, further analysis of the outcomes according to BP control patterns or antihypertensive medication status in these patients was limited. Third, cTnI levels were not measured in all patients, and it is likely that the cTnI test was performed in only relatively high-risk patients, so the possibility of selection bias cannot be excluded. In addition, we used only baseline cTnI values ​​and did not consider follow-up cTnI results. Fourth, data regarding ED revisit and readmission rates could have been underestimated. Finally, we could not identify cardiovascular events and mortality because the corresponding data of the cause of death were not provided by the National Health Insurance Service. However, data regarding all-cause mortality and date of death obtained from the National Health Insurance Service were highly accurate, as they cover the entire population of Korea. Further research is needed on the optimal screening, risk stratification, and treatment strategy as related to cardiovascular events or death according to troponin results in patients with hypertensive crisis.

In conclusion, the risk of all-cause mortality increased in patients with detectable cTnI and elevated cTnI levels, which was consistently observed regardless of the presence of acute HMOD. cTnI, which is an independent risk factor for all-cause mortality in patients with hypertensive crisis, might improve the prediction of mortality in patients with hypertensive crisis.

## Data Availability

The data that support the findings of this study are available from the corresponding author, JHS, upon reasonable request.
